# The Crystal Structure of OprG from *Pseudomonas aeruginosa*, a Potential Channel for Transport of Hydrophobic Molecules across the Outer Membrane

**DOI:** 10.1371/journal.pone.0015016

**Published:** 2010-11-29

**Authors:** Debra S. Touw, Dimki R. Patel, Bert van den Berg

**Affiliations:** Program in Molecular Medicine, University of Massachusetts Medical School, Worcester, Massachusetts, United States of America; Duke University, United States of America

## Abstract

**Background:**

The outer membrane (OM) of Gram-negative bacteria provides a barrier to the passage of hydrophobic and hydrophilic compounds into the cell. The OM has embedded proteins that serve important functions in signal transduction and in the transport of molecules into the periplasm. The OmpW family of OM proteins, of which *P. aeruginosa* OprG is a member, is widespread in Gram-negative bacteria. The biological functions of OprG and other OmpW family members are still unclear.

**Methodology/Principal Findings:**

In order to obtain more information about possible functions of OmpW family members we have solved the X-ray crystal structure of *P. aeruginosa* OprG at 2.4 Å resolution. OprG forms an eight-stranded β-barrel with a hydrophobic channel that leads from the extracellular surface to a lateral opening in the barrel wall. The OprG barrel is closed off from the periplasm by interacting polar and charged residues on opposite sides of the barrel wall.

**Conclusions/Significance:**

The crystal structure, together with recent biochemical data, suggests that OprG and other OmpW family members form channels that mediate the diffusion of small hydrophobic molecules across the OM by a lateral diffusion mechanism similar to that of *E. coli* FadL.

## Introduction

The Gram-negative bacterium *Pseudomonas aeruginosa* is an opportunistic human pathogen associated with lung infections in cystic fibrosis patients and nosocomial infections [1]. It has the ability to grow on a diverse range of carbon sources [2]. Like in other Gram-negative bacteria, the outer membrane (OM) creates an effective protective barrier to the permeation of small molecules [3]. Due to the impermeability of the OM, gram-negative bacteria have evolved three major classes of outer membrane proteins to facilitate the transport of nutrients into the cell: active transporters, general porins, and diffusion-driven specific transporters [2]. The TonB-dependent active transporters (*e.g*. FhuA and FepA in *E. coli*) are used for the uptake of large molecules such as iron-siderophore complexes [4], [5]. General porins (e.g. *E coli* OmpF) occur in many Gram-negative bacteria and form water-filled channels that facilitate the non-specific diffusion of small hydrophilic compounds across the outer membrane [6]. *P. aeruginosa* and other pseudomonads lack general porins and instead have a large number of substrate-specific channels for nutrient transport [2]. Due to the lack of porins, the OM of *P. aeruginosa* is highly impermeable, making it resistant to many antibiotics [1].

Besides small water-soluble compounds, the OM also forms an effective barrier against the permeation of hydrophobic molecules due to the presence of lipopolysaccharide (LPS) on the outside of the cell. The diffusion of many hydrophobic compounds across the OM is mediated by proteins belonging to the FadL family of OM channels [7]. FadL-mediated transport occurs via a mechanism involving lateral diffusion of the substrate from the barrel lumen, via an opening in the barrel wall, into the OM [8].

The OmpW family of small OM proteins is widespread among Gram-negative bacteria, with orthologs found in α, β, γ, and δ proteobacteria. Recent research in *Vibrio cholerae* has shown that OmpW is very immunogenic and present in all *V. cholerae* strains analyzed to date [9], [10], [11]. *E. coli* OmpW has been shown to be a receptor for colicin S4, which is part of the *E. coli* bacteriocin defense system [9], [12]. In addition, recent proteomic profiling studies have suggested a role for OmpW in osmoregulation [13]. However, none of these studies provid11/29/2010es any direct evidence for OmpW protein function. Perhaps the best clue for a putative function for OmpW family members is provided by sequence similarity to OM proteins present in operons dedicated to the biodegradation of small, hydrophobic molecules such as medium-chain alkanes (AlkL) and naphthalene (NahQ and DoxH) [14], [15], [16]. The crystal structure of *E. coli* OmpW showed an eight-stranded β-barrel with a hydrophobic channel containing a LDAO detergent molecule, supporting the notion that OmpW could mediate diffusion of hydrophobic molecules [17]. The channel present in *E. coli* OmpW is not a classical channel in the sense that it leads directly into the periplasm. Instead, the OmpW channel leads to a lateral opening in the barrel wall, suggesting a transport mechanism similar to that employed by FadL family members [8]. Very recently, OmpW from *Pseudomonas fluorescens* was shown to be required for the growth of this organism on naphtalene, the first direct evidence for function of a OmpW family member [18].

OprG is a major OM protein from *P. aeruginosa* and is an OmpW family member [19]. Its expression is very dependent on the growth conditions, suggesting a complex regulation. Decreased expression of OprG has previously been linked to increased antibiotic resistance, leading to speculation that OprG could be a transporter of norflaxin, tetracyclin and kanamycin [20]. Recently, OprG expression was found to be increased under high iron conditions when grown under anaerobic conditions [21]. Results for an *oprg* knockout strain, however, showed that OprG is not involved in iron or antibiotics uptake [21]. Very recently, OprG from *P. putida*, which is 70% identical to *P. aeruginosa* OprG, was shown to have a high emulsifying activity, leading to the suggestion that it may be involved in the utilization and uptake of hydrocarbons [22]. None of these studies, however, have provided a direct clue as to the function of OprG. For this reason we have determined the crystal structure of *P. aeruginosa* OprG at 2.4 Å resolution. The structure suggests that OprG forms a channel for the diffusion of small hydrophobic molecules.

## Results and Discussion

### Description of the overall structure

**Figure 1:**
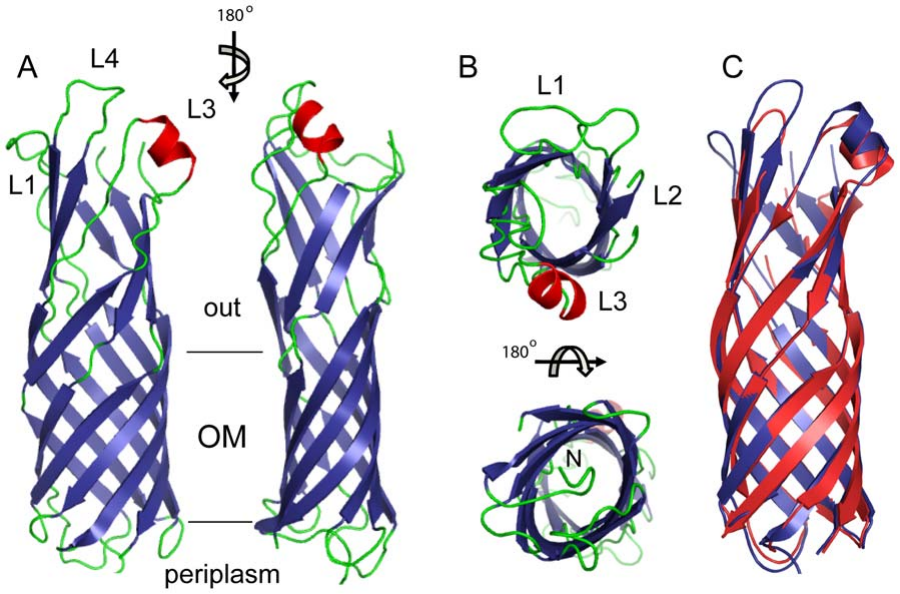
Structural overview of OprG. Views from the side (a) and from the extracellular side (b; top panel) and periplasmic side (b; bottom panel). β-strands are colored blue, α-helices red and loops green. Selected extracellular loops are indicated. The approximate positions of the outer membrane interface regions are indicated by horizontal lines. (c) Structural comparison between OprG (blue) and *E. coli* OmpW (red). Loops have been smoothed for clarity. This and the following figures were made with PYMOL [32].

OprG is structurally similar to *E. coli* OmpW (the C_α_ r.m.s.d. between OmpW and OprG is 0.9 Å; Fig. 1c). It forms an eight-stranded β-barrel about 50 Å in length with long extracellular loops (L) and short periplasmic turns (T) connecting the strands, as shown in Fig. 1. We observe density for the entire OprG molecule, with the exception of a short stretch of three residues (Gly80–Gly82) in the tip of loop L2, which are presumably disordered. The cross-section of the barrel has an oval shape with dimensions of ∼12×18 Å. In addition, OprG has a small α-helix in extracellular loop L3.

**Figure 2:**
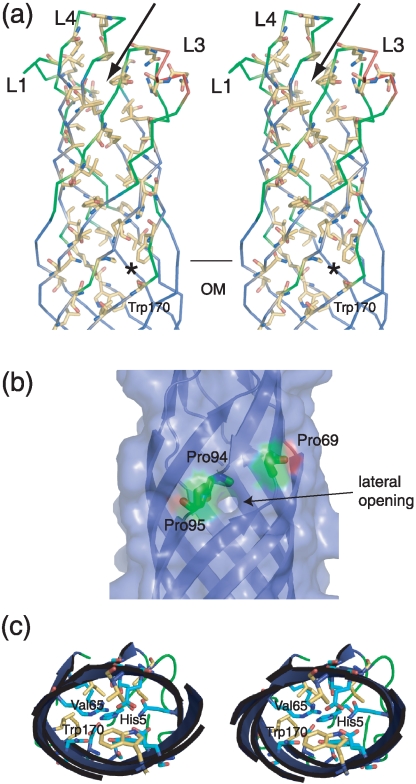
OprG has a distinct hydrophobic channel. (a) Stereoviews from the side, showing the amino acid residues of which the sidechains are pointing towards the barrel lumen (carbons yellow, oxygens red, nitrogens blue). A hydrophobic funnel on the extracellular side is indicated with an arrow. Residue Trp170 at the bottom of the hydrophobic channel is labeled. (b) Surface representation of the environment of the lateral opening. The location of the residues Pro69, Pro94 and Pro95 is shown. The lateral opening in the barrel wall is indicated by an arrow. (c) Stereoview from the extracellular side focusing on the lower (periplasmic) part of the barrel. Polar and charged residues that interact with each other to close the barrel are colored cyan, while hydrophobic residues are colored yellow. Residues Val65, Trp170 and His5 at the N-terminus are indicated.

**Figure 3:**
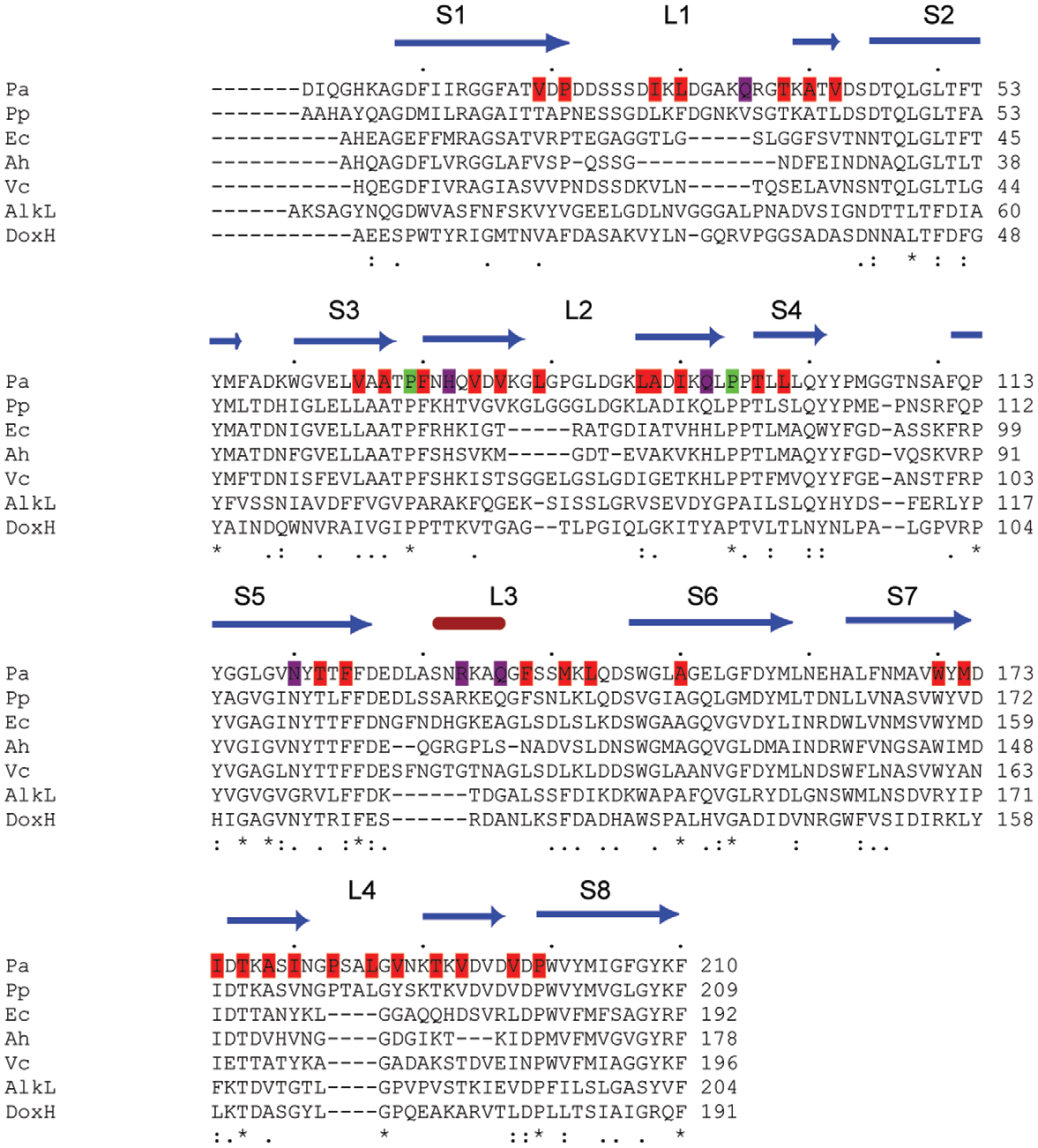
ClustalW alignment of OprG and other OmpW family members. The observed secondary structure of OprG is shown above the alignment, with β-strands (S) in blue and the α-helix in loop l3 in red. OprG residues are colored as follows: red; hydrophobic with sidechains pointing inwards, purple; polar/charged with sidechains pointing inwards and green; absolutely conserved prolines lining the lateral opening. The following orthologs have been aligned: Pa, *Pseudomonas aeruginosa* OprG; Pp, *Pseudomonas putida* OprG; Ec, *E. coli* OmpW; Ah, *Aeromonas hydrophila* OmpW; Vc, *Vibrio cholerae* OmpW; AlkL, *Pseudomonas oleovorans* AlkL; DoxH, *Pseudomonas* sp. (strain C18) DoxH.

A distinctive feature of OprG is that the lumen of the barrel on the extracellular side of the OM is lined almost exclusively with hydrophobic residues (Fig. 2a): of the ∼45 residues with sidechains pointing towards the lumen of the barrel, only ∼5 are polar (Gln35, His72, Gln92 Asn120, Arg133 and Gln136 in OprG). The many hydrophobic residues in the extracellular loops create a hydrophobic funnel that most likely forms a binding site for hydrophobic molecules (Fig. 2a). This notion is supported by the fact that although OprG does not have anything bound in this funnel, *E. coli* OmpW does have clear density for an LDAO detergent molecule at this position [17]. Interestingly, the hydrophobic character (but not the identity) of the residues lining the barrel lumen is conserved between members of the OmpW family (Fig. 3). The hydrophobic residues form a hydrophobic channel that extends approximately to the interface of the outer leaflet of the OM (Fig. 2a). At this position, Trp170 and Val65 come together to form the bottom of the channel. Thus, the hydrophobic channel does not continue all the way down into the periplasm. Interestingly, the residues on the periplasmic side of Trp170/Val65 are not hydrophobic, but predominantly polar and charged in character (Fig. 2c). A number of hydrogen bonds and salt bridges are present between these residues, similar to *E. coli* OmpA and OmpW [17], [23]. Both of these channels have been characterized in detail by single channel conductance experiments, showing that these small-diameter, apparently closed barrels can indeed form channels for the conductance of ions. However, the channels are not permanently open, and the observed conductance values are, as expected, very low (28 pS for OmpA and 19 pS for OmpW at 1 M KCl) [17], [24]. Thus, it seems unlikely that OprG can form permanently open channels with a conductance of ∼500 pS at 1 M KCl [21], a conductance value comparable to that of monomers of the porin OmpC [25].

### Comparison between OprG and other members of the OmpW family

An alignment of OmpW orthologs from five Gram-negative bacterial species (*Pseudomonas aeruginosa, Pseudomonas putida*, *Aeromonas hydrophila*, *Vibrio cholerae*, *Escherichia coli*) together with the more distantly related orthologs AlkL from *Pseudomonas oleovorans* and DoxH from *Pseudomonas* sp. C18 is shown in Fig. 3. The similarity among the different species, which range in sequence identity between ∼20% (AlkL, DoxH) and 70% (*P. putida*) compared to *P. aeruginosa* OprG, is greatest in the barrel region, with most divergence in the extracellular loops, something which is commonly observed for outer membrane proteins. Less than 15 residues are absolutely conserved across all species (Fig. 3); most of these are accounted for by glycines and outward-pointing (*i.e*. exposed to lipid) residues in the transmembrane regions, and are likely structurally important rather than functionally. Several absolutely conserved amino acids (Ala150, Thr176, Pro199) are hydrophobic residues with their sidechains pointing towards the barrel lumen, and are part of the hydrophobic channel. However, the most interesting conserved residues are Pro69 and Pro94. Both of these residues are adjacent to a lateral opening in the barrel wall between strands S3 and S4 (Fig. 2b). In addition, Pro95 is highly conserved, albeit not in more distantly related orthologs AklK and DoxH (Fig. 3). The prolines are likely responsible for the lateral opening, by interrupting the inter-strand hydrogen bond formation between strands S3 and S4. The conservation of the proline residues suggests that the lateral opening is present in all OmpW family members and thus may be functionally important. This notion is supported by the previously determined crystal structure of *E. coli* OmpW, which shows a lateral opening similar to that of OprG [17].

### A putative transport mechanism for OprG and other OmpW family members

**Figure 4:**
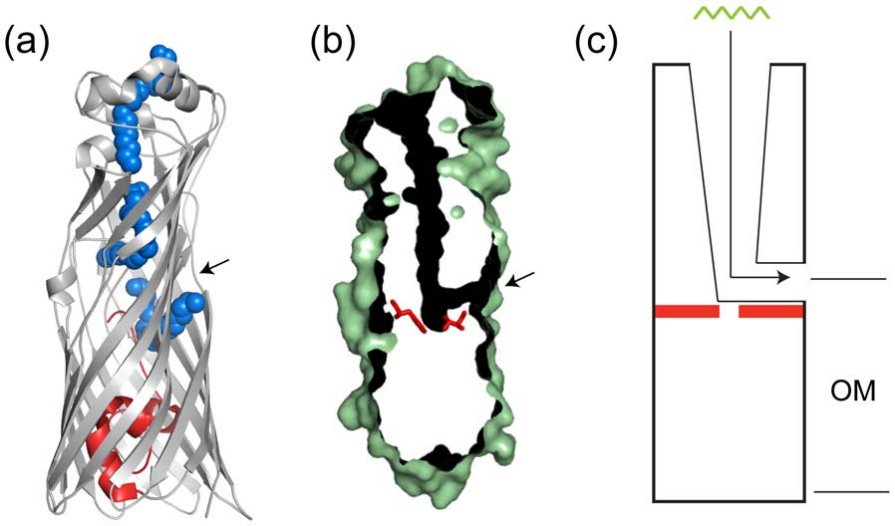
Proposed transport mechanism for OmpW family members. (a) Cartoon of *Pseudomonas aeruginosa* FadL (PDB ID: 3DWO) viewed from the side [8]. The hatch domain, closing off the barrel on the periplasmic side, is colored red. Bound detergent molecules delineating the hydrophobic transport channel are shown as space-filling models in blue. An arrow marks the lateral opening into the membrane. (b) Surface slab through the center of OprG, showing the hydrophobic channel as a dark tube. Residues Trp170 and Val65, forming the bottom of the channel, are shown in red. An arrow marks the lateral opening into the membrane. (c) Schematic model for transport of small hydrophobic substrates (depicted as octane in green) by members of the OmpW family. The bottom of the channel is shown in red.

The FadL family of OM channels is currently the only OM protein family that has been shown to be involved in the transport of hydrophobic molecules across the OM [7]. FadL channels form 14-stranded β-barrels and transport their substrates by lateral diffusion from the lumen of the barrel, through a lateral opening in the barrel wall, into the OM. Like FadL, OprG and other OmpW family members have a hydrophobic binding site on the extracellular side of the membrane (Fig. 2). In addition, the interior of the barrel is largely hydrophobic in both FadL and OmpW family members [8]. In FadL, a small N-terminal globular domain plugs the barrel on the periplasmic side, preventing direct diffusion of the substrates into the periplasm [26]. For OmpW proteins, the 8-stranded barrels are narrow enough so that interactions between polar/charged residues on opposite sides of the barrel wall effectively close the barrel towards the periplasm. Another similarity between FadL channels and OmpW proteins is the location of the lateral opening, approximately at the interface region of the outer leaflet of the OM. Thus, while clearly unrelated in sequence, OmpW proteins and FadL channels have the same structural features (Fig. 4), *i.e.* 1) a hydrophobic channel that runs from the extracellular surface to a lateral opening in the barrel wall and 2) a closure of the barrel lumen on the periplasmic side, effectively preventing the direct diffusion of substrates other than ions into the periplasm. Based on these structural arguments we propose that members of the OmpW family form channels for the uptake of small, hydrophobic molecules across the OM (Fig. 4). As for FadL family members, the final step in the diffusion process mediated by OmpW channels is likely to be the lateral diffusion of the substrate through a lateral opening in the barrel wall, into the OM. The next step will be to identify substrates for OmpW family members and to test the mechanism of transport in a similar way as has been done for *E. coli* FadL.

## Materials and Methods

The gene encoding for mature *P. aeruginosa* OprG was cloned from genomic DNA (ATCC) with EcoRI/XbaI restriction sites, digested and ligated into an arabinose inducible pB22 vector with the *E. coli* FadL signal sequence and an C-terminal histidine tag [27]. The tagged protein was over-expressed in C43 (DE3) cells via induction with 0.2% arabinose at 30°C for 5 hours. OprG was purified in a similar way as previously described for FadL [26]. The protein was concentrated to ∼10 mg/ml and dialyzed overnight against 10 mM sodium acetate pH 5.5, 50 mM NaCl, 10% glycerol, 0.4% C_8_E_4_. The final yield of purified protein was ∼0.3 mg per liter of cells. Initial crystallization trials were set up using Crystal Screen I (Hampton) and MembFac (Nextal). Small blocks were obtained in Crystal Screen condition # 82, and were optimized in 2–5 mM NiCl_2_/20% PEG 2K MME/0.1 M Tris pH 8.5. The crystals belong to space group I222 and diffract X-rays to about 2.3 Å resolution. There is one OprG molecule in the asymmetric unit (V_m_ = 2.52; 51% solvent). The data collection and refinement statistics are summarized in Table 1; the final model and structure factor have been deposited in the Protein Data Bank with accession code 2×27. Diffraction data were collected at NSLS (Brookhaven National Lab) Beamline X6A tuned to a wavelength of 1.1 Å. Images were processed in HKL2000 [28]. Phases were determined by molecular replacement using the program Phaser^17^ in the CCP4 software package^18^ with *E. coli* OmpW as the search model. The molecular replacement solution was then subjected to automated model building in Phenix [29]. Model manipulation in Coot [30], followed by further refinement in Phenix resulted in a final model with R_work_ = 0.191 and R_free_ = 0.268. The final model contains 12 C_8_E_4_ molecules, 3 nickel ions, and 113 water molecules. The entire sequence with the exception of residues 80–82 of L2 was built into electron density. Molprobity [31] found one Ramachandran outlier (Pro 94 at the lateral opening) that could not be refined to a preferred geometry. Although the Nickel K-edge is at ∼1.48 Å and the data were collected at 1.1 Å, processing the data with the anomalous flag on in HKL2000 resulted in an anomalous difference map with several peaks greater than 8 sigma (Ni f = 2.02 at 1.1 Å). The environments of the difference peaks are consistent with binding sites for a divalent cation. Taking into account the presence of 2 mM nickel in our crystallization condition we have assigned these anomalous peaks as nickel ions. Coordinates and phases have been deposited in the PDB with the accession code 2×27.

**Table 1:** Data collection and refinement statistics for OprG.

**Data Collection**	
Space Group	I222
Unit Cell Dimensions	
a, b, c (Å)	52.050, 58.380, 164.433
α, β, γ (°)	90, 90, 90
Resolution	40-2.40 (2.5-2.40)
R_merge_ (%)	10.5 (37.5)^1^
Completeness (%)	99.7 (96.4)
I/σ	7.4 (3.2)
Redundancy	4.7 (3.6)
**Refinement**	
Resolution Range (Å)	15-2.40
Total no. of Reflections	9990
Total no. atoms in refinement	
Protein	1615
C_8_E_4_	93
Nickel	3
Water	108
R_work_/R_free_ (%)	19.1/26.8
R.m.s. deviation from ideal	
Bond length (Å)	0.007
Bond angle (°)	1.094

1 Values in parentheses are for the highest resolution shell.
